# Effects of Mono-Vacancies and Co-Vacancies of Nitrogen and Boron on the Energetics and Electronic Properties of Heterobilayer h-BN/graphene

**DOI:** 10.3390/ma15186369

**Published:** 2022-09-14

**Authors:** Gladys Casiano Jiménez, Juan David Morinson-Negrete, Franklin Peniche Blanquicett, César Ortega-López, Miguel J. Espitia-Rico

**Affiliations:** 1 Grupo Avanzado de Materiales y Sistemas Complejos GAMASCO, Universidad de Córdoba, Montería CP 230001, Colombia; 2 Doctorado en Ciencias Física, Universidad de Córdoba, Montería CP 2030001, Colombia; 3 Grupo de Investigación AMDAC, Institución Educativa José María Córdoba, Montería CP 2300001, Colombia; 4 Grupo GEFEM, Universidad Distrital Francisco José de Caldas, Bogotá CP 110111, Colombia

**Keywords:** h-BN/graphene heterobilayer, Löwdin charge, magnetic metal, DFT

## Abstract

A study is carried out which investigates the effects of the mono-vacancies of boron (VB) and nitrogen (VN) and the co-vacancies of nitrogen (N), and boron (B) on the energetics and the structural, electronic, and magnetic properties of an h-BN/graphene heterobilayer using first-principles calculations within the framework of the density functional theory (DFT). The heterobilayer is modelled using the periodic slab scheme. In the present case, a 4 × 4-(h-BN) monolayer is coupled to a 4 × 4-graphene monolayer, with a mismatch of 1.40%. In this coupling, the surface of interest is the 4 × 4-(h-BN) monolayer; the 4 × 4-graphene only represents the substrate that supports the 4 × 4-(h-BN) monolayer. From the calculations of the energy of formation of the 4 × 4-(h-BN)/4 × 4-graphene heterobilayer, with and without defects, it is established that, in both cases, the heterobilayers are energetically stable, from which it is inferred that these heterobilayers can be grown in the experiment. The formation of a mono-vacancy of boron (1 V_B_), a mono-vacancy of nitrogen (1 V_N_), and co-vacancies of boron and nitrogen (V_BN_) induce, on the structural level: (a) for 1 V_B_, a contraction n of the B-N bond lengths of ~2.46% and a slight change in the interfacial distance D (~0.096%) with respect to the heterobilayer free of defects (FD) are observed; (b) for 1 V_N_, a slight contraction of the B-N of bond lengths of ~0.67% and an approach between the h-BN monolayer and the graphene of ~3.83% with respect to the FD heterobilayer are observed; (c) for V_BN_, it can be seen that the N-N and B-B bond lengths (in the 1 V_B_ and 1 V_N_ regions, respectively) undergo an increase of ~2.00% and a decrease of ~3.83%, respectively. The calculations of the Löwdin charge for the FD heterobilayer and for those with defects (1 V_B_, 1 V_N_, and V_BN_) show that the inclusion of this type of defect induces significant changes in the Löwdin charge redistribution of the neighboring atoms of VB and VN, causing chemically active regions that could favor the interaction of the heterobilayer with external atoms and/or molecules. On the basis of an analysis of the densities of states and the band structures, it is established that the heterobilayer with 1 V_B_ and V_BN_ take on a half-metallic and magnetic behavior. Due to all of these properties, the FD heterobilayer and those with 1 V_B_, 1 V_N_, and V_BN_ are candidates for possible adsorbent materials and possible materials that could be used for different spintronic applications.

## 1. Introduction

Graphene was obtained by Novoselov et al. [1] by means of mechanical exfoliation in 2004; it is a bidimensional material that possesses a hexagonal lattice and *sp*^2^ hybridation. Graphene is a semimetal with a zero band gap. Because of its excellent properties and wide range of applications, (microelectronics, optoelectronics, and nanoelectronics, among others), graphene has been extensively studied, both theoretically and experimentally. Nevertheless, one of the greatest obstacles to the construction of the new generation of graphene-based micro- and nanoelectronic devices is the absence of a finite band gap. Therefore, opening the band gap in graphene is currently a subject of interest. Enormous efforts have been made in pursuit of this goal, using various techniques; among them can be mentioned: quantum confinement by means of the reduction of its dimension by cutting the graphene into nanoribbons [2,3] or nanowires [4], use of graphene bilayers [5,6], twisted graphene [7], doping [8,9,10,11], graphene subjected to tension [12], hydrogenation [13], and growth of graphene on various substrates [14,15]. In all of these techniques that have been used, the two-carbon atom in a unit cell are not equivalent, generating a finite band gap in the graphene. However, the symmetry breaking of the graphene sublattices causes a significant increase in the effective mass of the charge carriers, which produces a large reduction in its mobility. Therefore, maintaining without changes in the effective mass of the charge carriers is very important. Because of this, opening the band gap and maintaining the high degree of mobility of the charge carriers in graphene is one of the most-researched topics in materials science. Various theoretical investigations have been carried out related to the heterobilayers of graphene with other materials [16,17,18,19,20,21,22,23,24,25,26,27,28,29,30]. In addition, various heterobilayers of graphene, along with other bidimensional materials, have been obtained [31,32,33,34,35,36,37,38,39,40,41,42,43], finding that they are potential candidates for the manufacture of new devices in optoelectronics and nanoelectronics based on graphene. In this sense, and due to the fact that a hexagonal BN monolayer (h-BN) has a structure similar to that of graphene (in both materials, the atoms possess a hexagonal arrangement in honeycomb form and their lattice constants differ by less than 2%), the graphene/BN [44,45,46,47,48,49,50,51,52,53] and that of BN/graphene heterobilayer [54,55,56,57,58,59,60,61,62,63] have been extensively studied. Some experimental studies reveal that graphene obtained by means of the aforementioned heterobilayers is of good quality and possesses a smooth morphology, free of bubbles or wrinkles, [64,65], and that the charge carriers have a high degree of mobility [64,65]. Lee et al. [66] found that the mobility of the charge carriers of graphene in the graphene/h-BN heterobilayer is three (3) times greater than that of graphene without h-BN. However, point defects can be present in any crystalline structure, with vacancies of atoms being the most frequent. Loh et al. [67], using the scanning tunneling microscopy technique, found discontinuities caused by vacancies of atoms in the graphene/h-BN heterobilayer, grown by means of the chemical vapor deposition technique. Recently, Neupename and Adhikari [68], using first-principles calculations, studied the effects of boron and nitrogen vacancies on the structural and electronic properties of a graphene/h-BN heterobilayer. However, a more thorough theoretical analysis and the knowledge of the effects of these vacancies on the electronic and energy properties of these heterobilayers is a problem that has not yet been completely resolved. A complementary study is crucial for an understanding of the functioning and performance of these graphene-based heterobilayers. Due to all of the above, in this paper, we present a detailed study of the effects that boron and nitrogen mono-vacancies and co-vacancies produce on the energetics (bonding energy, formation energy, and work of adhesion) and the electronic properties (electronic density of states (DOS), energy band structure, charge density, and Löwdin charge) of an h-BN/graphene heterobilayer. The results of the first investigations allow us to establish the intensity of the interactions between the BN and graphene monolayers and the stability of the BN/graphene heterobilayer and to infer the viability of its growth in the laboratory, while the results of the second studies allow us to perform a more complete analysis of the effects of B and N mono-vacancies and co-vacancies on the structural and electronic properties of a 4 × 4-(h-BN)/4 × 4-graphene heterobilayer.

## 2. Computational Method

The calculations were performed within the framework of density functional theory (DFT) [69,70], using Quantum ESPRESSO computational code [71,72]. The correlation and exchange potentials are modelled on the Perdew–Burke–Ernzerhof generalized gradient approximation (PBE-GGA) [73]. The external potentials are modelled by ultrasoft pseudopotentials. For the calculations, a plane-wave basis is used for the electronic orbitals and the electronic density with cutoff energies of 59 Ry and 500 Ry, respectively. The heterobilayers are modelled on the periodic slab scheme, considering vertical stacking of a 4 × 4-(h-BN) monolayer on a 4 × 4-graphene monolayer, considering a vacuum space of 20 Å in the z direction. The effects of the weak van der Waals (vdW) interaction between the monolayers of BN-h and graphene are included through the Grimme-D2 approximation [74]. The convergence criteria for the total energy, force, and pressure are 1 meV/atom, 1 meV/Å, and 0.2 kbar, respectively.

The analysis of the energy of the FD 4 × 4-(h-BN)/4 × 4-graphene heterobilayer through those with 1 V_B_, 1 V_N_, and V_BN_ was performed by means of the calculation of the bond energy (*E_b_*), the energy of formation (*E*_f_), and the work of adhesion (*W*_ad_), respectively. In order to avoid interactions between simultaneous vacancies of boron and nitrogen, a sufficiently large distance of 7.20 Å was used. The energy band diagrams (BD) and the total and projected densities of states (DOSs) for the principal electronic orbitals of the B, N, and C atoms were calculated in the irreducible zone of the first Brillouin zone (1 BZ) with a 14×14×1 Monkhorst–Pack mesh of k-points [75]. Finally, the study of the partial occupation of electronic states near the Fermi level (S_F_) was carried out by means of the Methfessel–Paxton method [76]. All the calculations were performed with spin-polarized calculations.

## 3. Results and Discussions

### 3.1. Strucural Parameters

Before beginning the study of the h-BN/graphene heterobilayer with and without a vacancy, we first carried out a complete structural optimization of the isolated h-BN monolayer and the isolated graphene. We found that, for the h-BN monolayer, the lattice constant was *a_BN_* = 2.5150 Å and the B-N bond length was *l_B-N_* = 1.4521 Å, while the lattice constant for the graphene was *a_gra_* = 2.4630 Å, and the C-C bond length was *l_C-C_* = 1.4200 Å. All these values are in excellent agreement with the results of previous theoretical [16,29,30,62,77,78,79,80,81,82] and experimental [1,83,84] investigations.

In order to investigate the effects of the mono-vacancies and co-vacancies of boron and nitrogen on the structural, electronic, and magnetic properties of an h-BN)/graphene heterobilayer, initially, we constructed a 4 × 4-(h-BN)/4 × 4-graphene heterobilayer, which contained 32 atoms of C, 16 atoms of B, and 16 atoms of N.

**Figure 1:**
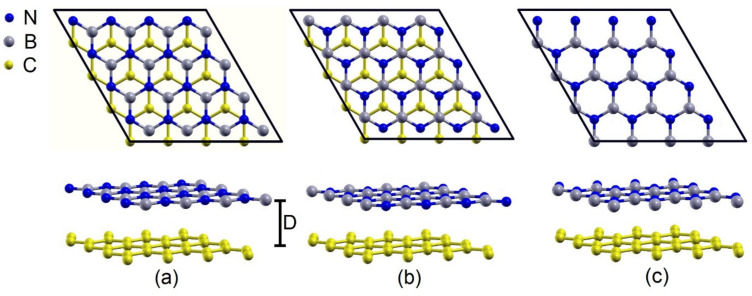
(4 × 4) BN-h (4 × 4)/graphene heterostructure (**a**) B-centered stacking, (**b**) N-centered stacking, and (**c**) h-centered stacking.

The 4 × 4-(h-BN)/4 × 4-graphene heterobilayer exhibits a mismatch of ~1.4%. In this configuration, three types of vertical stacking are considered: in the first stacking, an atom of B is exactly in the center of the graphene hexagon (B-centered); in the second stacking, an atom of N is exactly in the center of the graphene hexagon (N-centered); and, in the third stacking, the centers of the hexagons of h-BN and the graphene coincide, that is, the B and N atoms are exactly over the C atoms (h-centered), as is shown in Figure 1a–c, respectively.

**Figure 2:**
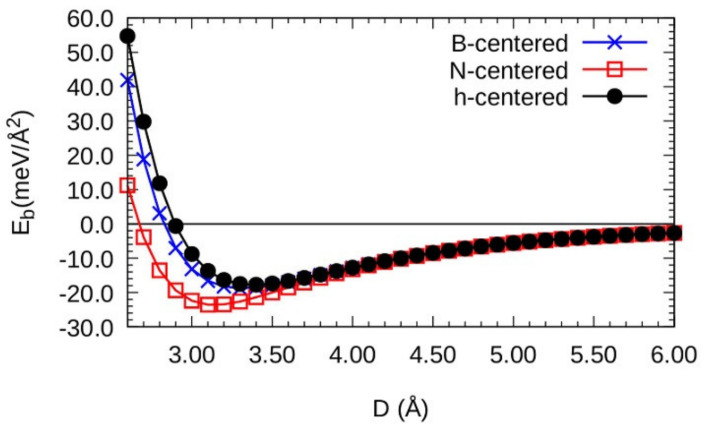
Bond energy (*E_b_*) vs. interfacial distance (D) for the vertical stacking h-BN/graphene heterobilayers; B-centered stacking (blue line and crosses), N-centered stacking (red line and squares), and h-centered stacking (black line and points).

In order to determine the energetically most favorable configuration for the three cases under consideration, we calculate the bond energy in the 4 × 4-(h-BN)/4 × 4-graphene heterobilayer. The bond energy (which is a measure of the strength of the interlayer interaction in layered materials) is calculated with the following equation [68,77]:

(1) $$E_{b}=\frac{E_{BN-h/graphene}-E_{BN-h}^{fix}-E_{Graphene}^{fix}}{A}$$

where $E_{BN-h/graphene}$ is the total energy of the relaxed h-BN/graphene heterobilayer; $E_{BN-h}^{fix}$ and $E_{graphene}^{fix}$ are the total energies of h-BN and graphene monolayers, respectively. For the calculation of $E_{BN-h}^{fix}$ and $E_{graphene}^{fix}$, the h-BN and graphene monolayers were maintained fixed in the positions in which they remained in the relaxed heterobilayer. A is the area of the upper horizontal plane of the heterobilayer. In Figure 2, the variation in the bond energy of the 4 × 4-(h-BN)/4 × 4-graphene heterobilayer is shown as a function of the distance of the interlayer separation D (the distance of the separation between the h-BN monolayer and the graphene) for the three kinds of stacking described in Figure 1. In Figure 2, the energetically most favorable configuration is that which has a nitrogen atom exactly in the center of the graphene hexagon, since this configuration has a minimum energy value (*E_b_* = −23.920 meV/Å), which corresponds to a distance of interlayer separation D = 3.1180. Hereafter, we refer to the energetically most favorable configuration, that is, the one that has a nitrogen atom exactly in the center of the graphene hexagon. This result is in excellent agreement with the results of the studies by Slotman et al. [85] in their investigation of dynamic stability by means of the calculation of phonons and by Giavannetti et al. [86] and Sachs et al. [87] in their studies of the energetic stability of the heterobilayer.

In Table 1, the values for the optimum structural parameters of the constituents that make up this heterobilayer (h-BN and graphene monolayers) are shown, together with the structural parameters of the isolated h-BN and graphene monolayers. It can be seen that, after the coupling process between the monolayers in order to form the N-centered h-BN/graphene heterobilayer, the h-BN contracts, with the decrease in the lattice constant and in the B-N bond length being ~1.11% and 1.12%, respectively, while the graphene expands, with the increase in the lattice constant and the bond length being ~0.98% and ~1.11%, respectively.

**Figure 3:**
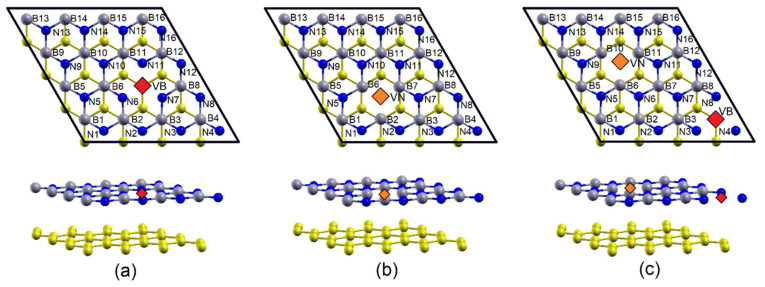
h-BN(4 × 4)/graphene (4 × 4) heterobilayer (**a**) 1 V_B_, (**b**) 1 V_N_, and (**c**) V_BN_, respectively.

In this subsection, the effects of the mono-vacancies of boron (1 V_B_) and nitrogen (1 V_N_), and the co-vacancies and simultaneous vacancies of boron and nitrogen (V_BN_), in an h-BN/graphene heterobilayer, are established. A schematic representation of these isolated defects is shown in Figure 3a–c, respectively. As can be seen, in Figure 3, each atom of B has three atoms of N as first neighbors and vice-versa (each atom of N has three atoms of B as first neighbors). Thus, when a vacancy of B is generated, its first neighbors (each atom of N) remain bonded to the other two atoms of B. Something similar occurs when a vacancy of N is generated: its first neighbor (each atom of B) remains bonded to the other two atoms of N, as shown in Figure 3a,b, respectively. With the aim of analyzing the effects of these vacancies on the bond length of the neighboring atoms of the vacancy, the atoms of B, as well as those of N, are labelled from 1 to 16.

Table 2 shows the B-N bond length (*l_B-N_*) and the interfacial distance D of the 4 × 4-(h-BN)/4 × 4-graphene heterobilayer with mono-vacancy and co-vacancy after the process of structural relaxation is finished. For 1 V_B_, we found that there exists a contraction of the B-N bond lengths of ~2.46% with respect to the heterobilayer with no vacancy, while, for 1 V_N_, there exists a slight contraction in the B-N bond lengths, with the maximum contraction being ~0.67%.

For the V_BN_, it is found that, in both the neighborhood of the B vacancy and in the neighborhood of the N vacancy, there occurs contractions in the B-N bond lengths, with the maximum values being ~3.62% and ~2.22%, respectively.

In relation to the interfacial distance D, we found that, in the heterobilayer with 1 V_B_, the interfacial distance practically does not undergo any change in the discrepancy with respect to the heterobilayer, with no vacancy being ~0.096%, while, for the heterobilayer with 1 V_N_, the monolayer of h-BN and the graphene undergo a significant approach, since the interfacial distance undergoes a contraction of ~3.83%.

On the other hand, for 1 V_B_, it is found that the distance N-N (between the three first neighbors of the B vacancy, atoms labelled with N6, N7, and N11 in Figure 3a) is *l_N-N_* = 2.5639 Å, which represents an increase of ~3.08%, with respect to the N-N length (*l_N-N_* = 2.4871 Å) in the heterobilayer free of vacancies, while, for 1 V_N_, the B-B distance (between the three first neighbors of the N vacancy, atoms labelled B2, B6, and B7 in Figure 3b), it is *l_B-B_* = 2.3563 Å, which represents a decrease of ~5.26% with respect to the B-B length (*l_B-B_* = 2.4871 Å) in the heterobilayer free of vacancies.

In relation to the V_BN_ covacancy, it is found that the N-N bond lengths (atoms labelled N3, N4, and N8 in Figure 3c) and B-B (atoms labelled B6, B7, and B11 in Figure 3c) are *l_N-N_* = 2.5366 Å and *l_B-B_* = 2.3917 Å, respectively; which corresponds to an increase of ~2.0% in the first case and a decrease of ~3.83% in the second one, compared to the same distances in the heterobilayer free of vacancies.

The increase in the N-N distance and decrease in the B-B distance indicate that the presence of 1 V_B_ and 1 V_N_ and the covacancy V_BN_ induce significant changes in the neighborhood of the vacancy in the structure of the h-BN monolayer. These results are in good agreement with the theoretical work of Huang and Lee [88] in their study of the effects of the B and N mono-vacancies in the hexagonal monolayer of h-BN.

With the aim of making a more complete study of the energy stability of the heterobilayer with B and N mono-vacancies and co-vacancies, we calculated the energy of formation and the work of adhesion. The energy of formation is defined as the energy needed to create the heterobilayer from its corresponding elemental forms. The energy of formation of the heterobilayer was calculated by means of the following equation [89,90,91,92]:

(2) $$E_{f}=\frac{E_{BN-h/grafeno}-E_{BN-h}^{iso}-E_{grafeno}^{iso}}{A}$$

where $E_{h-BN/graphene}$ is the total energy of the h-BN/graphene heterobilayer (FD, 1 V_B_, 1 V_N_, and V_BN_); $E_{BN-h}^{iso}$ and $E_{graphene}^{iso}$ are the total energies of the isolated h-BN monolayer (FD, 1 V_B_, 1 V_N_, and V_BN_) and of isolated graphene monolayer, respectively. A is the area of the heterobilayer.

Additionally, the work of adhesion was calculated, which is defined as the reversible work required to separate the heterobilayer into two free monolayers. In this case, the work of adhesion is defined as a negative value of the energy of formation [89,90], that is, W_ad_ = −E_for._ The values of the calculations of the bond energy, the energy of formation, and the work of adhesion for the heterobilayer free of defects, with mono-vacancies, and with B and N co-vacancies are shown in Table 3. In all of these cases, it is found that the energy of formation is negative; therefore, the heterobilayers are energetically stable, from which it can be inferred that they can be grown experimentally (exothermic processes).

### 3.2. Electronic Properties

To study the electronic properties of the h-BN/graphene heterobilayer free of vacancies, with 1 V_B_, 1 V_N_, and co-vacancy V_BN_, the density of states (DOSs), the band structure (BS), and the distribution of the density electronic charge of Löwdin were calculated. In the calculation of the DOSs and the BS, the Fermi level was chosen as the zero energy.

**Figure 4:**
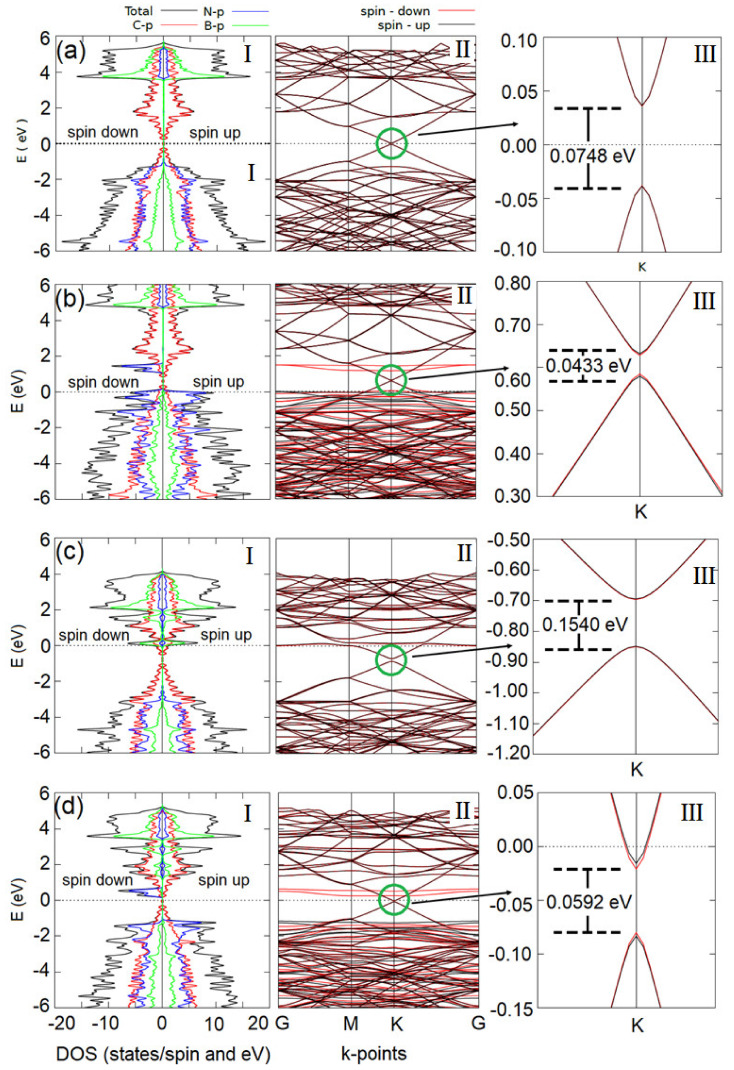
The BE and DOSs of the heterobilayer (**a**) without vacancy, (**b**) 1 V_B_, (**c**) 1 V_N_, and (**d**) co-vacancy V_BN_, respectively.

Figure 4a shows the DOSs and the BS of the h-BN/graphene heterobilayer free of vacancies. The BS (Figure 4a(II)) shows, at first sight, a behavior similar to that of the graphene, since the Dirac cone can be seen at point K, exactly at the Fermi level. This result is in excellent agreement with previous reports [90]. However, upon examining the BS exactly at the Fermi level, point K (Figure 4a(III)), it can be seen that the valence and conduction bands do not touch, that is, a finite band gap of 0.0748 eV = 74.8 meV appears. The value of this band gap is in good agreement with other values reported in the literature [93,94]. This band gap appears in the graphene due to the fact that the atoms of C interact with the non-homogeneous distribution of the charge density of the h-BN, making the C atoms non-equivalent, as the authors of references [93,94] explain in detail. Furthermore, in Figure 4a(I), it is shown that the contribution to the DOSs mainly comes from the p orbitals of the B, N, and C atoms. Finally, from Figure 4a(I), it is established that the heterobilayer free of defects does not possess magnetic properties, which is inferred from the symmetry of the states with spin up and spin down.

Figure 4b shows the DOSs and the BS of the h-BN/graphene heterobilayer with 1 V_B_. The BS (Figure 4b(II)) shows, at first sight, a behavior similar to that of the graphene, since the Dirac cone can be seen at point K, near the Fermi level. However, after closer inspection of the BS near the Fermi level, point K (Figure 4b(III)), it can be seen that there is a separation between the valence and the conduction bands, where a finite band gap of 0.0433 eV = 43.3 meV appears. Furthermore, in Figure 4bI, it is shown that the heterobilayer exhibits a half-metallic behavior. From Figure 4bI, it is established that the h-BN/graphene heterobilayer with 1 V_B_ has magnetic properties, which can be inferred from the asymmetry of the states with spin up and spin down of the valence band. The h-BN/graphene heterobilayer with 1 V_B_ acquires its magnetic properties mainly from the 2 p orbitals of the three N atoms that are bonded to the B atom that was removed in order to generate the vacancy. The value of the magnetic moment of the h-BN/graphene heterobilayer with 1 V_B_ is ~2.00 µ_β_/cell. The magnetic properties acquired by the heterobilayer through the boron vacancy open the door for the heterobilayer to be used in potential spintronic applications.

Figure 4c shows the DOSs and the BS of the h-BN/graphene heterobilayer with 1 V_N_. The BS (Figure 4c(II)), shows, at first sight, a significant separation of the Dirac cones at point K, near the Fermi level. After zooming in on the BS near the Fermi level, point K (Figure 4c(III)), a separation between the valence and the conduction bands can be seen, where a finite band gap of 0.1540 eV = 154.0 meV appears. The value of the band gap is approximately twice that of the heterobilayer free of defects. This increase in the finite band gap of the graphene in the heterobilayer represents a great improvement, since it favors the applications of the h-BN/graphene heterobilayer with 1 V_N_ in micro- and nano-electronic devices. In addition, the mono-vacancy of N in the heterobilayer does not induce magnetic effects in the hetero bilayer, which is verified by the symmetry of the density of states with spin up and spin down of the DOSs (Figure 4c(I)).

Figure 4d shows the DOSs and the BS of the h-BN/graphene heterobilayer with V_BN_ co-vacancies. The BS (Figure 4d(II)) shows, at first sight, a behavior similar to that of the graphene, since the Dirac cone can be seen at point K, near the Fermi level. However, after examining the BS near the Fermi level, point K (Figure 4b(III)), it can be seen that the valence and the conduction bands do not touch; that is, a separation of the Dirac cones occurs, and a finite band gap of 0.0592 eV = 59.2 meV, appears. Furthermore, in Figure 4d(I), it is shown that the heterobilayer exhibits a half-metallic behavior. From Figure 4d(I), it is established that the h-BN/graphene heterobilayer with a V_BN_ co-vacancy has magnetic properties, which is inferred from the asymmetry of the states with spun up and spin down of the valence band. The h-BN/graphene heterobilayer with V_BN_ co-vacancy acquires its magnetic properties mainly from the 2 p orbitals of the three N atoms that are bonded to the B atom that was removed in order to generate the vacancy. The value of the magnetic moment of the h-BN/graphene heterobilayer with co-vacancy V_BN_ is ~2.00 µ_β_/cell. The magnetic properties acquired by the heterobilayer through the boron and nitrogen co-vacancy open the door for potential applications in spintronics.

**Figure 5:**
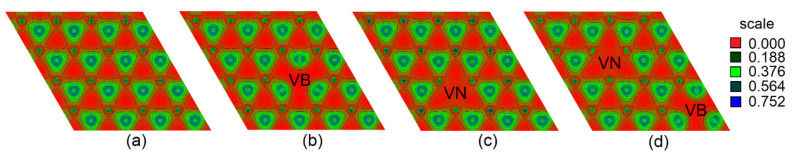
Contour maps of the electronic density of the heterobilayer (**a**) without vacancy, (**b**) 1 V_B_, (**c**) 1 V_N_, and (**d**) co-vacancy V_BN_, respectively.

In order to complete the analysis of the effect of the mono-vacancies and co-vacancies of boron and nitrogen on the electronic properties of the heterobilayer, we calculated the total electronic charge distribution and per Löwdin orbital of the atoms in the h-BN/graphene heterobilayer free of defects, with a B vacancy (1 V_B_), with an N vacancy (1 V_N_), and with a co-vacancy of nitrogen and boron (V_BN_). The results are shown in Table 4 and Figure 5 (distribution of the electronic charge density). As can be seen in the scale of colors of Figure 5, blue indicates a great electronic charge density in these regions; therefore, the N atoms possess a greater charge density than the B atoms in the heterobilayer. This result is confirmed by the total Löwdin charge per N atom, listed in Table 4. It can be seen in Table 4 that, when a boron vacancy is generated (1 V_B_) in the heterobilayer, there is a slight decrease in the total Löwdin charge in the neighboring atoms of the vacancy (nitrogen N6, N7, and N11; Figure 3a); on the other hand, due to the greater negative charge density of these three nitrogen atoms, together with the absence of the B atom, the repulsion between them shoots up, which explains the increase in the N-N separation distance between the N6, N7, and N11 atoms of ~3.08%. In addition, the calculations of the Löwdin charge density per orbital for the N6, N7, and N11 atoms show that the s-N electronic orbitals undergo, on average, an increase in the electronic charge of 0.116 e/Bohr^3^, while the p-N orbitals undergo, on average, a decrease in the electronic charge of 0.237 e/Bohr^3^. This last result is coherent with the appearance of the peaks with spin down associated mainly with the p-N states and p-B in minor contribution in the DOSs (Figure 4b(I)). The ~2.00 µ_β_/cell magnetic moment induced by the B mono-vacancy is associated with the redistribution and transfer of charge from the graphene to the h-BN monolayer. This result is in excellent agreement with the calculations by Park et al. [95], where a detailed explanation of this charge transfer is given.

In the same way, in Figure 5b, a contour map of the distribution of the electronic charge density associated with the h-BN/graphene heterobilayer with 1 V_B_, significant changes can be seen in the forms of the electronic densities associated with the first-neighbor atoms (N6, N7, and N11) of the boron vacancy.

It can be seen in Table 4 that, when a nitrogen mono-vacancy (1 V_N_) is generated in the heterobilayer, there is a slight increase in the total Löwdin charge in the neighbor atoms of the vacancy (atoms B2, B6, and B7 Figure 3b). However, in spite of this slight charge increase, atoms B2, B6, and B7 experience an attraction, which explains the reduction in the B-B distances between atoms B2, B6, and B7 of 5.26%. Furthermore, as is shown in Table 4, the calculations of the Löwdin charge density per orbital for atoms B3, B6, and B7 show that the s-B electronic orbitals experience, on average, a decrease in the electronic charge of 0.083 e/Bohr^3^, while the p-B orbitals experience, on average, an increase in the electronic charge C. This last result is coherent with the appearance of the peaks associate with the p-B states, near the Fermi level in the DOSs (Figure 4c(I)).

Finally, it can be seen in Table 4 that, when a co-vacancy of boron and nitrogen (V_BN_) is generated in the heterobilayer, a behavior similar to that described for the mono-vacancies B and N is found. For the atoms B6, B10, and B11 (neighbors of the nitrogen vacancy 1 V_N_), the total Löwdin charge undergoes, on average, an increase of 0.104 e/Bohr^3^, the average increase in the Löwdin charge (per orbital) in the orbitals s-B and p-B are 0.087 e/Bohr^3^ and 0.017 e/Bohr^3^, respectively, while, in the atoms N3, N4, and N8 (neighbors of 1 V_B_), the total Löwdin charge undergoes, on average, a decrease of 0.091 e/Bohr^3^.

The calculation of the Löwdin charge density (per orbital) shows that the s-N orbitals of atoms N3, N4, and N8 present an average increase of 0.017 e/Bohr^3^, while the p-N orbitals of the atoms N3, N4, and N8 experience an average decrease of 0.198 e/Bohr^3^. These results are coherent with the contour map of Figure 5d, which shows the changes in the forms of the electronic densities associated with atoms B2, B6, B7, N3, N4, and N8, respectively.

## 4. Conclusions

The effects of mono-vacancies and co-vacancies of B and N on the energetics and electronic properties of the 4 × 4-(h-BN)/4 × 4-graphene heterobilayer were investigated using the GGA-PBE-D2 generalized gradient approximation and atomic pseudopotentials. Initially, from the calculations of the energetics, it was established that the heterobilayers’ FD, with boron vacancy 1 V_B_, nitrogen vacancy 1 V_N_, and co-vacancy of boron and nitrogen V_BN_, are energetically stable. Furthermore, it was found that the inclusion of 1 V_B_ and 1 V_N_ in the 4 × 4-(h-BN)/4 × 4-graphene heterobilayer induces slight structural changes in the distance between the monolayers of 4 × 4-(h-BN) and 4 × 4-graphene and in the bond length (B-N, B-B, and N-N) of the neighbor atoms of the 1 V_B_ and 1 V_N_, respectively.

From the analysis of the Löwdin charge transfer for the 4 × 4-(h-BN)/4 × 4-grafene heterobilayers’ FD and with 1 V_B_, 1 V_N_, and V_BN_, it was established that this type of point defect generates significant changes in the distribution of the Löwdin charge in the first-neighbor atoms of the VB and VN, respectively; these local zones are converted into chemically active regions that could favor interaction between the surfaces of these heterobilayers with external atoms and/or molecules.

From the analysis of the density of states and the band structure, it was established that the inclusion of 1 V_B_ and 1 V_BN_ in the 4 × 4-(h-BN)/4 × 4-graphene heterobilayer induces, in both cases, a half-metallic and magnetic behavior in this heterobilayer, from which it is inferred that the la 4 × 4-(h-BN)/4 × 4-graphene heterobilayer could be used in spintronic applications.

## Figures and Tables

**Table 1:** Optimum structural parameters for the h-BN and graphene monolayers obtained in the N-centered h-BN/graphene heterobilayer and optimum structural parameters for the isolated h-BN and isolated graphene monolayers.

	a (Å)	*l*_B-N_ (Å)	*l_C_*_-C_ (Å)
h-BN in heterobilayer	2.4871	1.4357	-
Graphene in heterobilayer	2.4870	-	1.4359
Isolated h-BN	2.5150	1.4521	-
Isolated graphene	2.4630	-	1.4200

**Table 2:** Principal structural parameters measured in Å: BN bond lengths ($l_{Ni-Bj}$) and interfacial distances (D). The labels *i* and *j* correspond to the neighbor atoms of the vacancies. The prime superscripts (′) correspond to the images of the atoms.

Heterostructure VB	$l_{B2-N6}$(Å)	$l_{B6-N6}$(Å)	$l_{B11-N11}$(Å)	$l_{B12-N12}$(Å)	$l_{B3-N7}$(Å)	$l_{B8-N7}$	D (Å)
	1.4003	1.4003	1.4003	1.4003	1.4003	1.4003	3.1150
Heterostructure VN	$l_{B2-N1}$(Å)	$l_{B2-N2}$(Å)	$l_{B6-N5}$(Å)	$l_{B6-N10}$(Å)	$l_{B7-N7}$(Å)	$l_{B7-N11}$(Å)	D (Å)
	1.4265	1.4264	1.4263	1.4261	1.4266	1.4267	2.9986
Heterostructure VBN	$l_{B16^{\prime }-N4}$(Å)	$l_{B1\prime -N4}\left(Å\right)$	$l_{B15\prime -N3}$(Å)	$l_{B3-N3}$(Å)	$l_{B8-N8}$(Å)	$l_{B5\prime -N8}$(Å)	D (Å)
Neighbors a VB	1.4025	1.4025	1.3837	1.3978	1.3978	1.3837	3.1203
	$l_{B6-N5}$(Å)	$l_{B6-N6}$(Å)	$l_{B10-N9}$(Å)	$l_{B10-N14}$(Å)	$l_{B11-N15}$(Å)	$l_{B11-N11}$(Å)	D (Å)
Neighbors a VN	1.4038	1.4173	1.4133	1.4133	1.4038	1.4173	3.1100

**Table 3:** Energetics in the h-BN/graphene heterobilayer, FD, and with 1 V_B_, 1 V_N_, and V_BN_, respectively. The energy is given in units of meV/$\;{Å}^{2}$.

Energetics	$E_{b}\;(\mathrm{meV}/\;{Å}^{2})$	*E_f_* $\left(\mathrm{meV}/\;{Å}^{2}\right)$	*W_sep_* $\;(\mathrm{meV}/\;{Å}^{2})$
Heterobilayer FD	−23.92	−21.00	21.00
Heterobilayer VB	−25.43	−22.17	22.17
Heterobilayer VN	−29.59	−29.95	29.95
Heterobilayer VBN	−24.46	−22.81	22.81

**Table 4:** Electronic charge per Löwdin orbital (measured in units of electrons e/Bohr^3^) h-BN/graphene heterobilayer with vacancy and without 1 V_B_, 1 V_N_, and V_BN_, where Q_t_ is total charge, Q_s_ is the charge in the s orbital, and Q_p_ is the total charge in the p orbital.

Atoms	Without Vacancy			1 V_B_			1 V_N_			V_NB_		
	Q_t_	Q_s_	Q_p_	Q_t_	Q_s_	Q_p_	Q_t_	Q_s_	Q_p_	Q_t_	Q_s_	ΔQ_p_
B1	2.524	0.611	1.913	2.536	0.613	1.923	2.516	0.602	1.914	2.541	0.612	1.929
B2	2.524	0.611	1.913	2.553	0.609	1.944	2.650	0.694	1.956	2.518	0.614	1.904
B3	2.524	0.611	1.913	2.553	0.609	1.944	2.516	0.603	1.913	2.542	0.607	1.935
B4	2.524	0.611	1.913	2.536	0.613	1.923	2.516	0.606	1.910	----	----	----
B5	2.524	0.611	1.913	2.533	0.613	1.920	2.509	0.612	1.897	2.545	0.602	1.943
B6	2.524	0.611	1.913	2.553	0.609	1.944	2.649	0.694	1.955	2.630	0.698	1.932
B7	2.524	0.611	1.913	----	----	----	2.650	0.694	1.956	2.519	0.603	1.916
B8	2.524	0.611	1.913	2.553	0.609	1.944	2.509	0.612	1.897	2.542	0.607	1.935
B9	2.524	0.611	1.913	2.533	0.609	1.924	2.508	0.609	1.899	2.517	0.614	1.903
B10	2.524	0.611	1.913	2.533	0.609	1.924	2.509	0.612	1.897	2.623	0.698	1.925
B11	2.524	0.611	1.913	2.553	0.609	1.944	2.516	0.603	1.913	2.630	0.698	1.932
B12	2.524	0.611	1.913	2.553	0.609	1.944	2.509	0.612	1.897	2.518	0.614	1.904
B13	2.524	0.611	1.913	2.533	0.613	1.920	2.509	0.612	1.897	2.518	0.611	1.907
B14	2.524	0.611	1.913	2.533	0.609	1.924	2.509	0.612	1.897	2.517	0.614	1.903
B15	2.524	0.611	1.913	2.533	0.613	1.920	2.516	0.606	1.910	2.545	0.602	1.943
B16	2.524	0.611	1.913	2.536	0.613	1.923	2.516	0.606	1.910	2.541	0.612	1.929
N1	5.421	1.194	4.227	5.410	1.204	4.206	5.397	1.197	4.200	5.408	1.199	4.209
N2	5.421	1.194	4.227	5.418	1.195	4.223	5.398	1.197	4.201	5.415	1.202	4.213
N3	5.421	1.194	4.227	5.410	1.204	4.206	5.415	1.189	4.226	5.332	1.299	4.033
N4	5.421	1.194	4.227	5.410	1.199	4.211	5.415	1.189	4.226	5.326	1.304	4.022
N5	5.421	1.194	4.227	5.410	1.204	4.206	5.398	1.197	4.200	5.403	1.201	4.202
N6	5.421	1.194	4.227	5.304	1.310	3.994	----	----	----	5.405	1.197	4.208
N7	5.421	1.194	4.227	5.295	1.310	3.985	5.398	1.198	4.200	5.421	1.192	4.229
N8	5.421	1.194	4.227	5.410	1.204	4.206	5.414	1.194	4.220	5.332	1.299	4.032
N9	5.421	1.194	4.227	5.417	1.195	4.222	5.408	1.191	4.217	5.399	1.205	4.194
N10	5.421	1.194	4.227	5.418	1.195	4.223	5.397	1.197	4.200	----	----	----
N11	5.421	1.194	4.227	5.297	1.310	3.987	5.397	1.197	4.200	5.405	1.197	4.208
N12	5.421	1.194	4.227	5.418	1.195	4.222	5.408	1.191	4.217	5.415	1.202	4.213
N13	5.421	1.194	4.227	5.418	1.195	4.223	5.408	1.191	4.217	5.410	1.194	4.216
N14	5.421	1.194	4.227	5.417	1.195	4.222	5.414	1.194	4.220	5.399	1.205	4.194
N15	5.421	1.194	4.227	5.410	1.204	4.206	5.415	1.189	4.226	5.403	1.201	4.202
N16	5.421	1.194	4.227	5.410	1.204	4.206	5.414	1.194	4.220	5.408	1.199	4.209

## Data Availability

The data obtained in this research are unpublished and are not listed in any databases.
